# Fully Integrated 24-GHz 1TX-2RX Transceiver for Compact FMCW Radar Applications

**DOI:** 10.3390/s24051460

**Published:** 2024-02-23

**Authors:** Goo-Han Ko, Seung-Jin Moon, Seong-Hoon Kim, Jeong-Geun Kim, Donghyun Baek

**Affiliations:** 1 School of Electrical Engineering, Chung-Ang University, Seoul 06974, Republic of Korea; rngks79@cau.ac.kr (G.-H.K.); alex97msj@cau.ac.kr (S.-J.M.); tjdgns123@cau.ac.kr (S.-H.K.); 2 Department of Electronic Engineering, Kwangwoon University, Seoul 01897, Republic of Korea; junggun@kw.ac.kr

**Keywords:** CMOS, FMCW radar, FMCW synthesizer, sensor, transceiver

## Abstract

A fully integrated 24-GHz radar transceiver with one transmitter (TX) and two receivers (RXs) for compact frequency modulated continuous wave (FMCW) radar applications is here presented. The FMCW synthesizer was realized using a fractional-N phase-locked loop (PLL) and programmable chirp generator, which are completely integrated in the proposed transceiver. The measured output phase noise of the synthesizer is −80 dBc/Hz at 100 kHz offset. The TX consists of a three-bit bridged t-type attenuator for gain control, a two-stage drive amplifier (DA) and a one-stage power amplifier (PA). The TX chain provides an output power of 13 dBm while achieving <0.5 dB output power variation within the range of 24 to 24.25 GHz. The RX with a direct conversion I-Q structure is composed of a two-stage low noise amplifier (LNA), I-Q generator, mixer, transimpedance amplifier (TIA), a two-stage biquad band pass filter (BPF), and a differential-to-single (DTS) amplifier. The TIA and the BPF employ a DC offset cancellation (DCOC) circuit to suppress the strong reflection signal and TX-RX leakage. The RX chain exhibits an overall gain of 100 dB. The proposed radar transceiver is fabricated using a 65 nm CMOS technology. The transceiver consumes 220 mW from a 1 V supply voltage and has 4.84 mm^2^ die size including all pads. The prototype FMCW radar is realized with the proposed transceiver and Yagi antenna to verify the radar functionality, such as the distance and angle of targets.

## 1. Introduction

In recent years, the demand to sense the surrounding environment has been rapidly increasing, not only in the automotive industry but also in appliance businesses, such as smart appliances, energy control, security, and emergency detection, with the development of the Internet of Things (IoT). Sensor types used in various applications include radar, laser, camera, infrared, and ultrasonic [1,2]. Among them, industrial, scientific, and medical (ISM)-band 24 GHz radar based on frequency-modulated continuous wave (FMCW) is in the spotlight because it is not sensitive to weather, light condition, and temperature, and can be installed invisibly [3,4]. However, FMCW radar systems have lacked competitiveness owing to their complex structure and high bill of materials (BOM) compared to other sensors. For example, an FMCW radar system being implemented by combining discrete radio frequency integrated chips (RFICs) on a printed circuit board (PCB) has large size, high cost, and high system complexity. In order to solve these problems, several works have been established to integrate discrete integrated chips (ICs), such as a transmitter (TX), a receiver (RX), and local oscillator (LO), into a single chip [5,6,7,8,9]. However, although the previous works have unique advantages in terms of the number of TX-RX channels and power consumption, there are still considerable shortcomings regarding the size and cost-effectiveness of FMCW radar applications. In [5,6], multi-channel TX-RX radar transceivers were presented for the FMCW radar. In [7,8], 1-TX and 1-RX radar transceivers with TRX leakage cancellation and the TRX novel switching technique were presented for a single antenna operation. However, they still require external ICs such as phase-locked loop (PLL) for chirp generation and analog baseband for intermediate frequency (IF) amplification. This constrains their usage in compact and low-cost FMCW radar applications. Another requirement for the FMCW radar is a fast chirp generation capability. In [9], a fully integrated 1TX and 2-RX radar transceiver was presented using complementary metal oxide semiconductor (CMOS) technology. However, the slow-chirp waveform with ramp duration > 1 ms was used in a target detection scenario. A radar using the slow chirp has range-doppler ambiguity and poor signal-to-noise ratio (SNR) in the IF signals because the IF signals suffer from the baseband flicker noise and the noise floor of the other targets. Thus, a fast chirp is required to improve these problems. The fast chirp is makes it possible to move the IF signal of the targets away from the flicker noise corner of the baseband and increase the frequency difference between the IF signals in a multi-target detection scenario [10,11]. 

In this paper, a fully integrated 24 GHz 1TX-2RX transceiver for compact FMCW radar applications is presented. An FMCW synthesizer being integrated in the proposed transceiver generates the fast chirp waveform through a fractional-N PLL, voltage-controlled oscillator (VCO), and programmable chirp generator. The FMCW synthesizer supports triangular and sawtooth waveforms with reconfigurable pulse repetition internal (PRI). The TX chain exhibits a high output power by utilizing a two-stage driving amplifier (DA) and a one-stage power amplifier (PA) with a common-source (CS) structure including a neutralization capacitor. Furthermore, the 0–7 dB attenuation stage for TX gain control is realized through a fully differential bridged T-type attenuator. The RX chain with a direct conversion I-Q structure demonstrates high linearity at the given chirp bandwidth by adopting the transformer-based gain-bandwidth enhancements technique. An analog baseband in the RX chain mitigates the problem of TX-RX leakage and short-range interference through a range compensation filter with a −40 dB slope. 

The remainder of this paper is organized as follows. Section 2 presents the fundamental principle of the FMCW radar. Section 3 provides the system architecture and the details of circuit implementations of key building blocks. Further, Section 4 verifies the transceiver and radar functionality with the measurement results. Finally, Section 5 concludes this article.

## 2. FMCW Radar Principle

**Figure 1:**
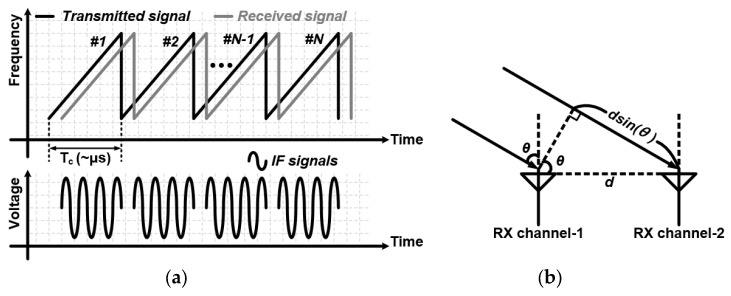
(**a**) FMCW radar principle and (**b**) angle estimation based on the monopulse phase comparison method.

The FMCW radar transmits a continuous wave, whose frequency changes linearly, and receives the signal reflected from the targets. The FMCW radar compares the received signal to the transmitted signal by mixing them. Then, IF signals are extracted due to the time delay between two signals, as shown in Figure 1a. After the extracted IF signals are sampled by the analog-to-digital converter (ADC), 2-D fast Fourier transform (FFT) is performed to obtain information about the range and velocity of the targets [12,13].

An FMCW radar consisting of two receivers determines the angle of the targets using the mono-pulse phase comparison method, as shown in Figure 1b. The angle is determined by comparing the phase difference between the received signals from two separate antenna arrays, as given by Equation (1):

(1) $$\theta =\sin^{-1}[\frac{ʎ\cdot (\varphi_{RX1}-\varphi_{RX2})}{2\pi \cdot d}]\;$$

where ʎ is wavelength, $\varphi_{RX1}$ and $\varphi_{RX2}$ are the phases of the IF signals for the targets, and *d* is the distance between the antenna arrays, respectively.

## 3. Design of 24 GHz 1TX-2RX FMCW Radar Transceiver

**Figure 2:**
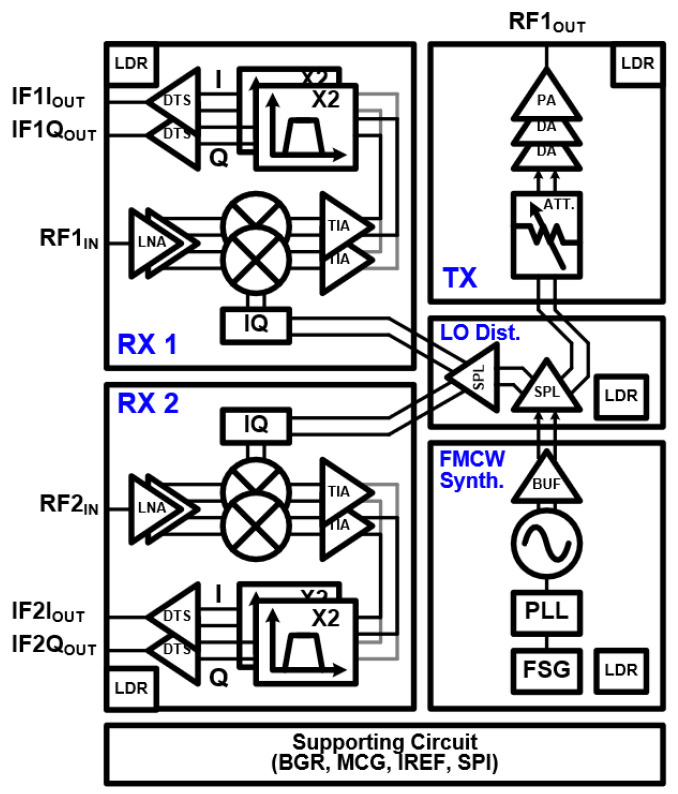
Block diagram of the proposed 24 GHz 1TX-2RX FMCW radar transceiver.

The proposed transceiver consists of a one-channel TX, two-channel RXs, LO and supporting circuits such as a bandgap reference (BGR), a master current generator (MCG), a reference current generator (IREF), low-dropout regulators (LDRs), and a serial–peripheral interface (SPI) as shown in Figure 2. The FMCW synthesizer consists of a fractional-N PLL, a class-C VCO operating at 24 GHz, a buffer to drive the VCO output, and a frequency sweep generator (FSG) for the programmable chirp generation. The buffer that drives the VCO output provides 24 GHz differential signals to the first split buffer in the LO distribution network. The first split buffer output is connected to the second split buffer input to distribute the signals to the two-channel receiver, and is connected to the attenuator input in the TX. The TX includes three-bit attenuator for gain control with an attenuation step of 1 dB, two-stage DA and one-stage PA. Each RX includes a two-stage LNA, a passive LC I/Q generator, a mixer, a trans-impedance amplifier (TIA), and an analog baseband. The analog baseband consists of a two-stage biquad band pass filter (BPF) and a differential-to-single amplifier (DTS).

### 3.1. FMCW Synthesizer

**Figure 3:**
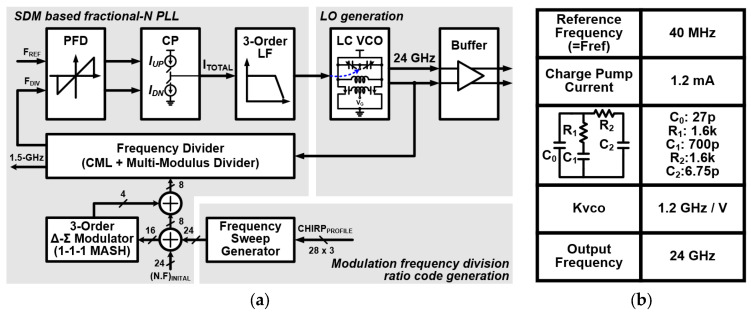
(**a**) Block diagram and (**b**) parameter values of the FMCW synthesizer.

**Figure 4:**
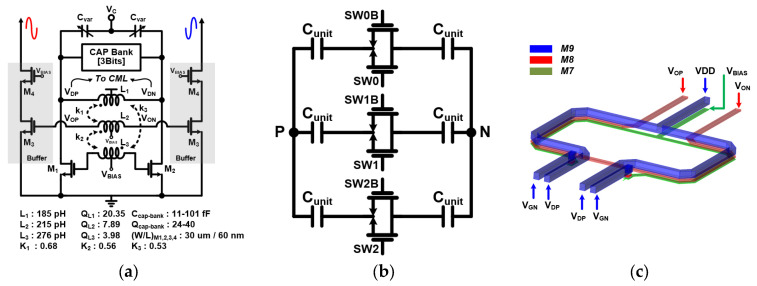

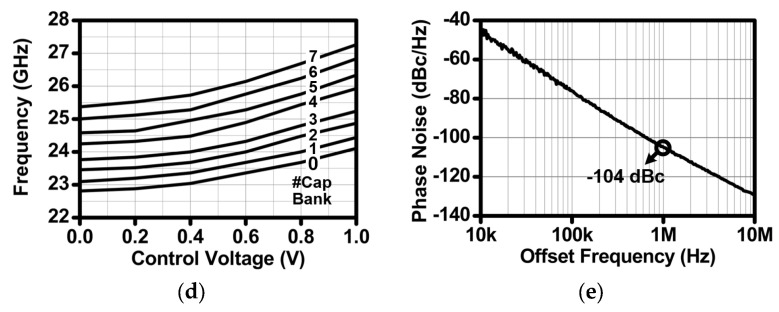
(**a**) Detailed schematics and parameter values of the VCO with the driving buffer, (**b**) the schematic of the three-bit cap bank, (**c**) layout of the triple-coupled transformer, (**d**) measured frequency range, and (**e**) measured phase noise.

The overall block diagram and parameter values of the FMCW synthesizer are shown in Figure 3. The FMCW synthesizer consists of three parts: a sigma-delta modulator (SDM)-based fractional-N PLL, an LC VCO for LO generation, and a FSG. The SDM-based fractional-N PLL is composed of a phase frequency detector (PFD), a charge pump (CP), a three-order loop filter (LF), a frequency divider stage, and a three-order multistage noised-shaping (MASH) SDM for fractional division. The frequency divider stage is realized through a fixed 1/4 and 1/16 current mode logic (CML) divider and an eight-bit multi-modulus divider (MMD). The fixed 1/4 CML divider provides the VCO output frequency divided by 4 to the MMD. The LC VCO for the LO generation is implemented with a triple-coupled transformer for the class-c bias technique, a three-bit cap bank for a wide frequency operation range, and a mos-varactor. The detailed schematics and parameter values of the VCO with the driving buffer are described in Figure 4a. Each inductor L1 and L2 is connected to the CML and the driving buffer, respectively. Figure 4b,c show the schematic of the three-bit cap bank and the layout of the triple-coupled transformer, respectively. The cap bank includes three T-gate switches and unit capacitors arrays. The triple-coupled transformer consists of three top metals. All transformers are stacked in a 1:1 transformer configuration. Figure 4d,e shows the measurement results of the LC class-c VCO. The measured frequency-tuning range of the VCO is 22.8–27.3 GHz. Further, the phase noise is −104 dBc/Hz at 1-MHz offset frequency.

**Figure 5:**
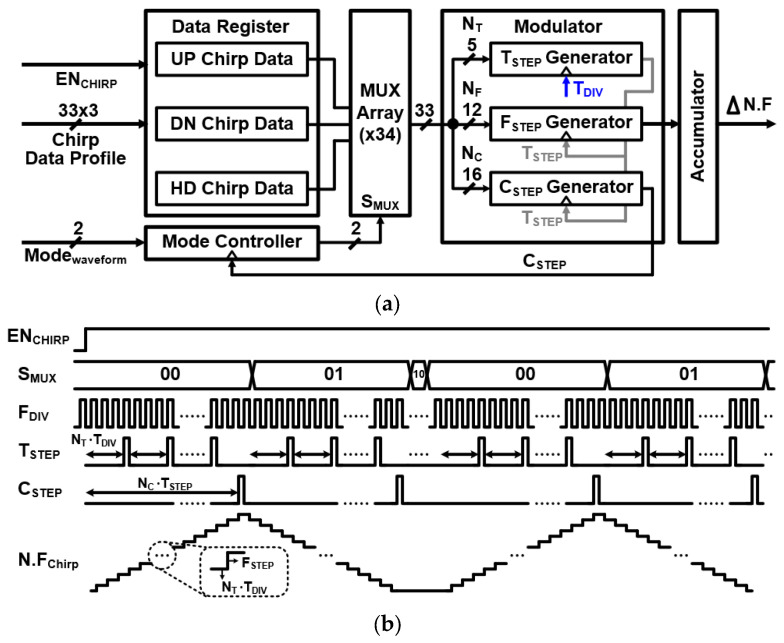
(**a**) Block diagram and (**b**) timing diagram of the FSG.

The FSG generates a programmable modulated frequency division ratio code. The input of the FSG consists of 33-bit data, which determine a FMCW waveform parameter such as a chirp time and a chirp bandwidth. The output of the FSG consists of 24-bit data. The lower 16-bit controls the fractional division ratio (FDR) of the SDM, and the upper 8-bit controls the integer division ratio (IDR) of the MMD. When the FDR exceeds the integer 1-division ratio, the IDR of the MMD is increased to the 1-division ratio while maintaining the extra FDR exceeding the 1-division ratio. As a result, the FSG provides ΔN.F at the modulation start frequency, being determined as N.F_initial_. Figure 5 shows a detailed block diagram and timing diagram of the FSG.

The FSG consists of a mode controller, a data register, a multiplexer array, a modulator, and an accumulator. The mode controller, implemented as a finite state machine, determines the chirp sequence according to the two-bit input code. It has three modes: 00 (Hold chirp), 01 (Up chirp–Down chirp), and 10 (Up chirp–Down chirp–Hold chirp). The data register has three datasets, which determine the FMCW waveform parameter. Each dataset is structured in a 33-bit format, which allocates 5 bits for the unit time step (N_T_), 12 bits for the unit frequency step (N_F_), and 16 bits for the Up-, Down-, and Hold-chirp period steps (Nc). The multiplexer array provides corresponding 33-bit data to the modulator depending on the mode. The modulator includes a T_STEP_ generator, a F_STEP_ generator, and a C_STEP_ generator. The T_STEP_ generator determines a clock T_STEP_ (= T_DIV_N_T_) to provide a reference clock to the F_STEP_ generator and the C_STEP_ generator. The F_STEP_ generator determines a frequency variation F_STEP_ (= F_RES_N_F_) to be changed according to T_STEP_, where the F_RES_ is the resolution frequency of the PLL. Finally, the C_STEP_ generator determines a clock C_STEP_ (= T_STEP_N_C_) to shift the chirp sequence. Therefore, the chirp bandwidth BW is determined by the frequency variation F_STEP_ and N_C_ as BW = F_STEP_ × N_C_.

### 3.2. Transmitter

**Figure 6:**
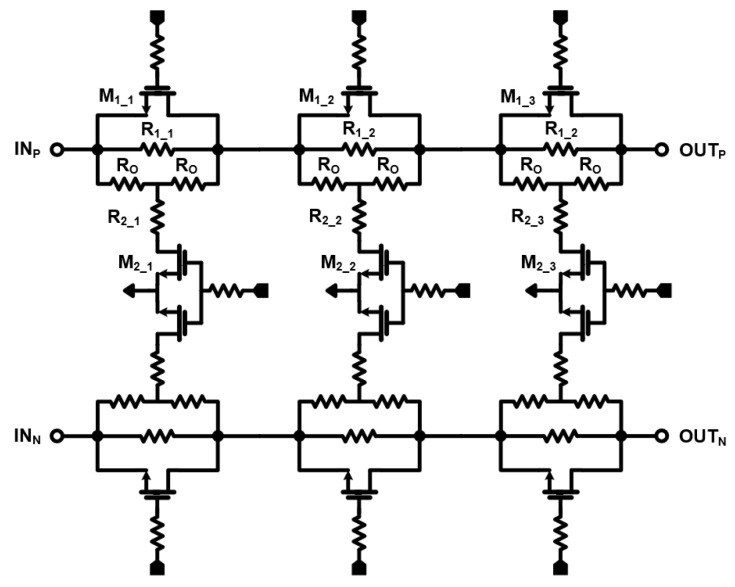
Schematic of the three-bit attenuator.

**Figure 7:**
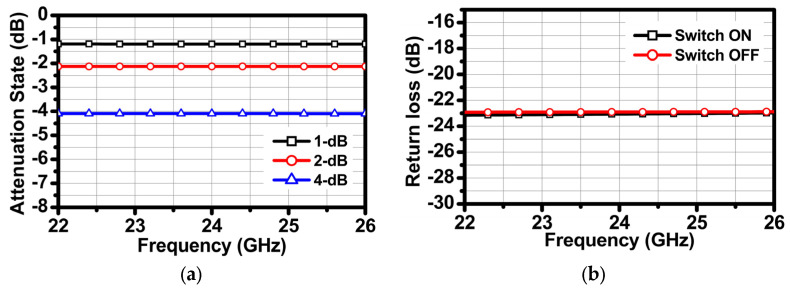
Simulation results: (**a**) relative attenuation state and (**b**) return loss according to switch state.

The three-bit attenuator is designed as a differential bridged T-type for low mismatch and high linearity. A body floating technique is adopted to improve the insertion loss and prevent signal leakage. The schematic of the three-bit attenuator is depicted in Figure 6. *R_1_* and *R_2_* are determined by Equations (2) and (3) where *R_O_* is the characteristic impedance and ATT_dB_ is the desired attenuation in the dB scale. The attenuation values of each stage are 1 dB, 2 dB, and 4 dB. Figure 7 shows simulation results for the relative attenuation state and return loss according to the switch state.

(2) $$R_{1}=R_{O}(10^{\frac{{ATT}_{dB}}{20}}-1)$$

(3) $$R_{2}=\frac{R_{O}}{\left(10^{\frac{{ATT}_{dB}}{20}}-1\right)}$$

**Figure 8:**
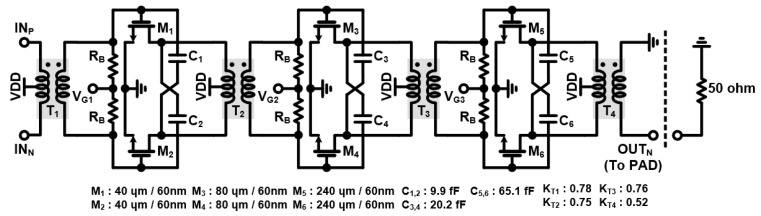
Schematic of the two-stage DA and one-stage PA.

**Figure 9:**
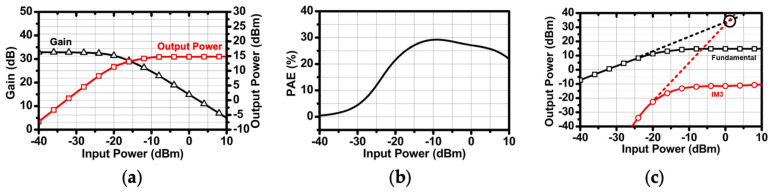
Simulation results of the DA and PA: (**a**) gain and Pout versus Pin, (**b**) PAE, and (**c**) IP3.

Figure 8 shows the overall schematic of the two-stage DA and one-stage PA. All stages adopt the transformers (T_1_, T_2_, T_3_, T_4_) to improve bandwidth. The topology of the DA and PA is designed as a CS amplifier with a neutralization capacitor. The neutralization capacitor is a capacitor that crosses a differential signal and acts as a negative capacitor because the phase of the differential signal is reversed. This cancels the parasitic gate-drain capacitance of the transistor, increasing the input impedance of the CS amplifier and eliminating the negative feedback path. In conclusion, linearity and isolation can be increased, and the maximum available gain (MAG) value can be improved by reducing S_12_. In addition, since the amplifier consists of one stack, unlike the cascode structure, the V_OV_ consumption generated by the transistor is reduced and the output voltage swing is increased. The load impedance of the PA is optimized to the impedance that can transmit the highest output power through the load pull test under the conditions of the 50 ohm output port. The simulated optimal load pull impedance is (21 + j11) ohm. Figure 9 summarizes the simulation results of the overall stage, achieving a gain of about 32.5 dB, an output P1 dB of 13.8 dBm, a power-added efficiency (PAE) of 29.3%, and an output three-order intercept point (IP3) of about 34.5 dBm. 

### 3.3. Receiver

**Figure 10:**
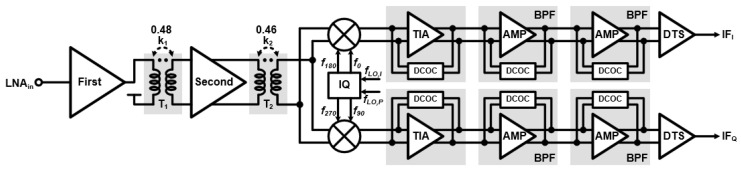
Overall architecture of the RX.

**Figure 11:**
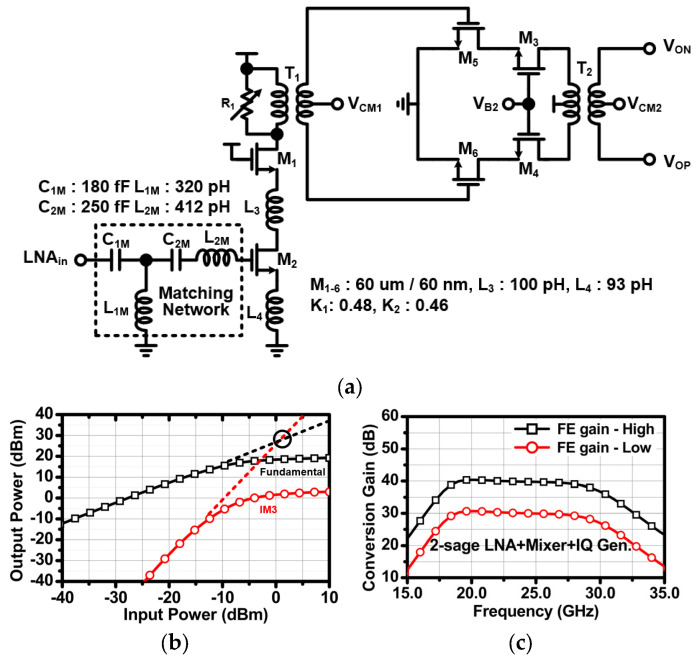
(**a**) Schematic and (**b**) simulation results of the two-stage LNA, and (**c**) front end conversion gain without TIA.

The Figure 10 shows the overall architecture of the RX. The RX consists of a transformer-based two-stage LNA, a mixer, an IQ generator, a TIA, a two-stage BPF, and a DTS amplifier. The two-stage LNA adopts the transformer-based gain-bandwidth enhancement technique to realize wideband gain at the given chirp bandwidth. The wideband gain is generated from a magnetically coupled resonator with an output capacitance of the preceding stage’s LNA, the input capacitance of the next stage’s LNA, and the low coupling factor k of the transformer. This magnetically coupled resonator exhibits wide impedance over two resonance points depending on capacitance and transformer values. Figure 11a shows the schematic of the two-stage LNA. The first stage LNA is implemented with a single-input cascode amplifier topology, including a matching inductor L_1_, a source degeneration inductor L_2_, and variable resistor R_1_ for gain control. The input impedance of the first-stage LNA matches 50 ohm through an LC matching network. The matching network employs two capacitors (C_1M_ and C_2M_) and two inductors (L_1M_ and L_2M_). The second stage’s LNA is implemented with a differential cascode amplifier topology. The two transformers (T_1_ and T_2_) are optimized so that the gain of the front end has wideband characteristics. Figure 11b shows the simulation results of the two-stage LNA. When the two-stage LNA operates in high gain mode, it achieves an input P1 dB of −15.5 dBm and an input IP3 of 0 dBm. The mixer is a conventional double-balanced passive mixer [14], and the IQ generator is an LC-type poly-phase filter [15]. The I-Q LO signals drive the gate of the passive mixers, and the LNA output current signals are amplified through the TIA. In Figure 11b, the front-end conversion gain demonstrates wideband gain from 19 to 28 GHz, which is sufficient to cover the chirp bandwidth. The TIA is implemented with a DC offset cancellation circuit (DCOC), which cancels the voltage DC offset and forms a high-pass filter (HPF) with −20 dB slope. Likewise, the HPF of the two-stage BPF in analog baseband is also implemented as DCOC. The TIA and first-stage BPF create the HPF region with −20 dB slope at the same cut-off frequency. This can achieve an HPF with a total of −40 dB slope, which mitigates the problem of TX–RX leakage and short-range interference. The second-stage BPF forms the HPF region with −20 dB slope at the cut-off frequency of 156 Hz to further suppress TX to RX leakage. The cut-off frequency of the HPF with −40 dB slope is controlled using two-bit to realize the variation (68, 125, 250, and 500 kHz). The cut-off frequencies of the low-pass filter (LPF) of the TIA and the two-stage BPF are the same at 2 MHz. This establishes an LPF region with a total of 60 dB slope.

(4) $$H(s)=\frac{R_{F}}{R_{I}}\cdot \frac{s\frac{1}{R_{F}C_{F}}}{s^{2}+s\frac{1}{R_{F}C_{F}}+{\left(\frac{1}{\sqrt{R_{C}R_{D}C_{D}C_{F}}}\right)}^{2}}$$

(5) $$H_{HPF}(s)=\frac{s}{s+\frac{R_{F}}{C_{D}R_{C}R_{D}}}$$

**Figure 12:**
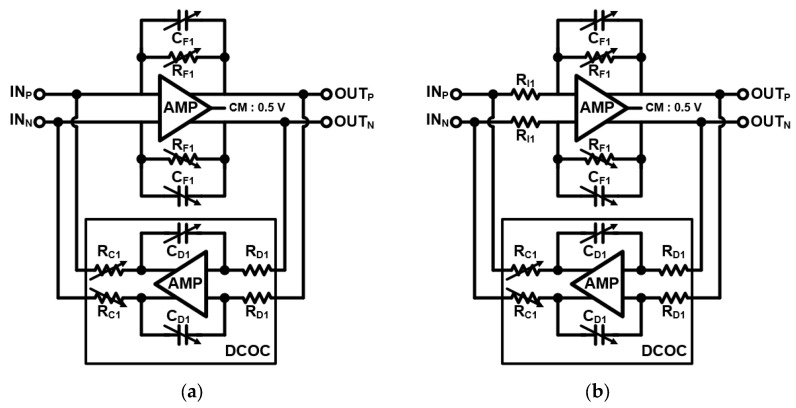
(**a**) Schematic of the TIA with DCOC and (**b**) schematic of the one-stage BPF.

**Figure 13:**
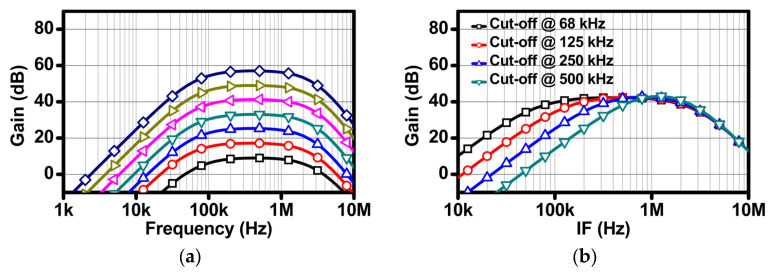
Simulation results: (**a**) gain variation and (**b**) the HPF cut-off frequency variation.

From the transfer function given by Equations (4) and (5), we can see that the gain of the two-stage BPF is the ratio of *R_F_* and *R_I_* and the LPF cut-off frequency is determined by *R_F_* and *C_F_*. The *HPF* cut-off frequency is determined by *C_D_*, *R_F_*, *R_C_*, and *R_D_*. Figure 12 shows a detailed schematic of the TIA and the one-stage BPF. The circuits employ the differential op-amps. The common-mode feedback (CMFB) of the op-amp is included in the op-amp itself. The phase margin of the CMFB in the op-amp is designed to be > 50 degrees, and the phase margins for TIA and BPF including the DCOC circuits are also secured to be more than 50 degrees. The simulation results of the TIA and two-stage BPF are summarized in Figure 13. Figure 13a shows the gain variation of the TIA and two-stage BPF. The receiver achieves the maximum gain of the TIA and BPF stages of 60 dB, and the controllable gain step is 1 dB. Figure 13b shows the HPF cut-off frequency variation induced by two-bit code control when the gain is 40 dB. 

## 4. Measurement Results

### 4.1. Transceiver

**Figure 14:**
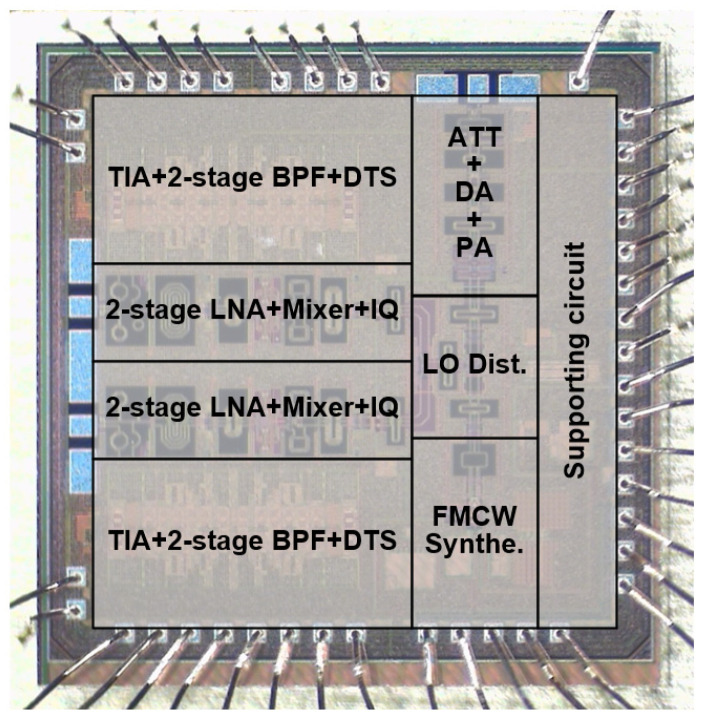
Chip microphotograph.

The chip microphtograph of the proposed 1TX-2TX transceiver is shown in Figure 14. The chip size, including all pads, is 4.84 mm^2^. A 2.5 V supply voltage is applied to the internal LDRs, which provide the 0.9–1.2 V internal voltages to each sub-block circuit. All analog and power pads are protected from electrostatic discharge (ESD) using the gate-coupled MOSFET and clamp circuits. Table 1 shows the current consumption of each block. The total current consumption is 220 mA when all the block is turned on in the FMCW radar mode. 

The proposed radar transceiver is measured using an on-chip measurement setup, wherein the transceiver chip is glued onto the PCB. The radar functionality is measured using a radar module including the proposed transceiver and Yagi antenna. 

**Figure 15:**
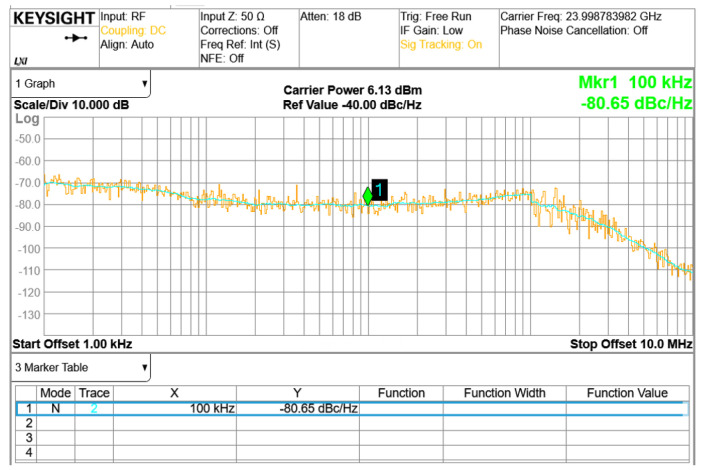
Measured phase noise at 24 GHz carrier frequency.

**Figure 16:**
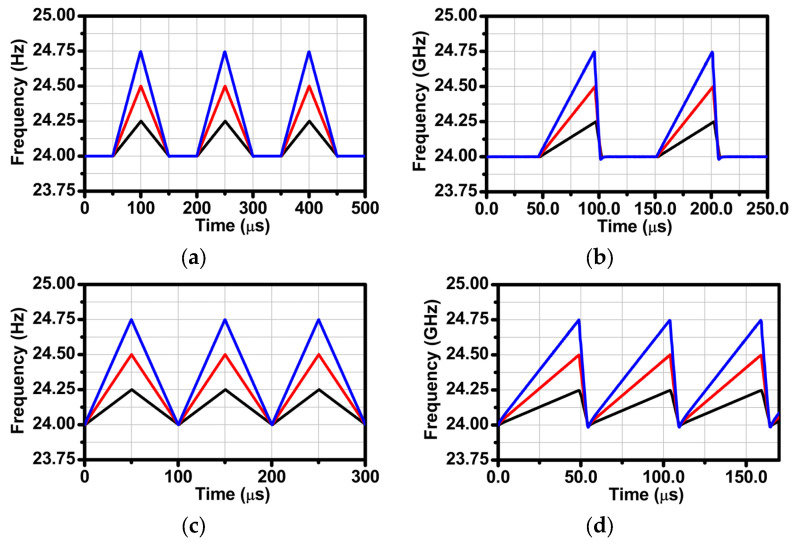
Measured chirp transient when chirp bandwidth is 250 (black line), 500 (red line), and 750 (blue line) MHz: (**a**) triangular waveform with hold-chirp time, (**b**) sawtooth waveform with hold-chirp time, (**c**) triangular waveform without hold-chirp time, and (**d**) sawtooth waveform without hold-chirp time.

The phase noise performance is shown in Figure 15. The phase noise for the 24 GHz carrier frequency is −80 dBc/Hz, with an offset frequency of 100 kHz when using a 40 MHz crystal as the reference frequency. Figure 16 demonstrates the measured chirp transient of triangular waveforms and sawtooth waveforms using a signal source analyzer in the time domain. The difference between Figure 16ab–cd relates to whether the hold-chirp time is set or not. 

**Figure 17:**
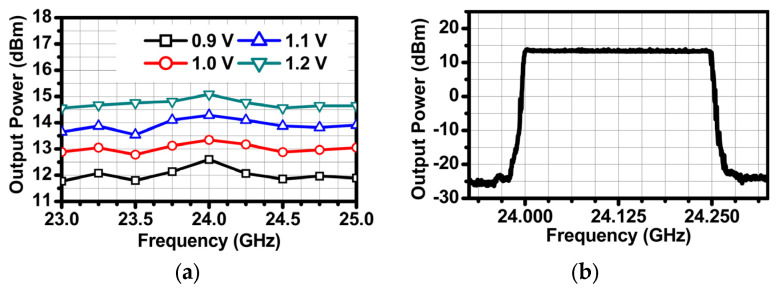
Measurement results: (**a**) output power versus the operation frequency and (**b**) chirp spectrum.

The TX and RX were measured with the on-wafer measurement method using a ground–signal–ground (GSG) probe. The total calibrated loss due to the GSG probe, RF connector and cable is 4 dB. The measured output power of the TX reaches 13.3 dBm when the supply voltage is 1 V, provided by the LDR. The output power can be reconfigured by changing the supply voltage, as shown in Figure 17a. The measured output power variation in the chirp spectrum is less than 1 dB within the range of 24 to 24.25 GHz, as shown in Figure 17b.

**Figure 18:**
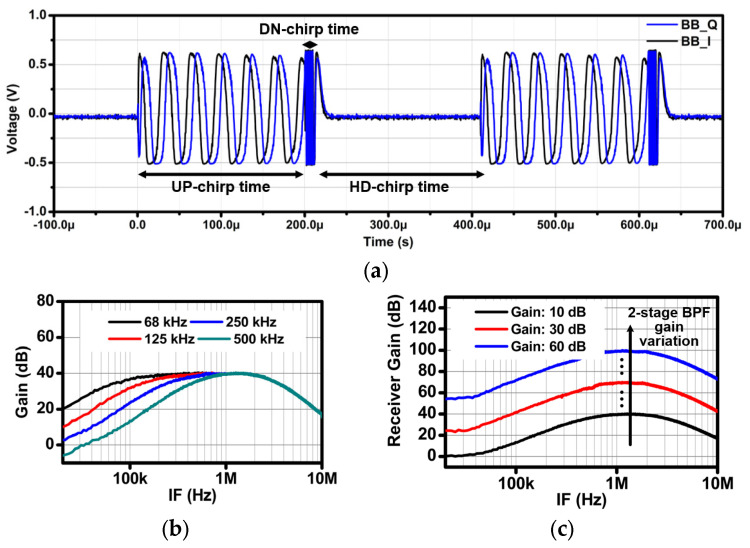
Measurement results: (**a**) TRX loopback test, (**b**) the HPF cut-off frequency variation, and (**c**) gain variation when the HPF cut-off frequency was 500 kHz.

The RX was measured through the output port of the DTS amplifier in the time domain and frequency domain to verify its functional operation. First, a loopback measurement was performed to check the voltage waveform of the down-converted IF signals. The proposed transceiver was operated in the FMCW mode. The TX output port links to the LNA input port of the RX through a cable. Subsequently, IQ IF signals corresponding to the cable length are generated as output. Figure 18a shows the measurement results for the parameter set at an Up-chirp time of 200 μs, a chirp bandwidth of 250 MHz, and a cable length of 4 m. The frequency of the IF signal is about 33 kHz. When substituted into the radar range equation, this frequency corresponds to a distance of 7.92 m. Figure 18b,c demonstrate the HPF cut-off frequency variation with a fixed gain of 40 dB, and gain variation with a fixed HPF cut-off frequency of 500 kHz. The signal generator was set to generate a 24 GHz signal, which was then directed to the RX while maintaining a fixed output power. Likewise, the proposed transceiver was operated in the continuous wave (CW) mode at 24 GHz. Then, alterations in the HPF cut-off frequency and gain were controlled through SPI. The output of the RX was observed with a spectrum analyzer in the frequency domain. The measured HPF cut-off frequencies were about 68, 125, 250, and 500 kHz according to the two-bit control code, while maintaining the LPF cut-off frequency at about 2 MHz. The maximum measured RX conversion gain was about 100 dB. A comparison of performance with previous works is summarized in Table 2.

### 4.2. Radar Functionality

**Figure 19:**
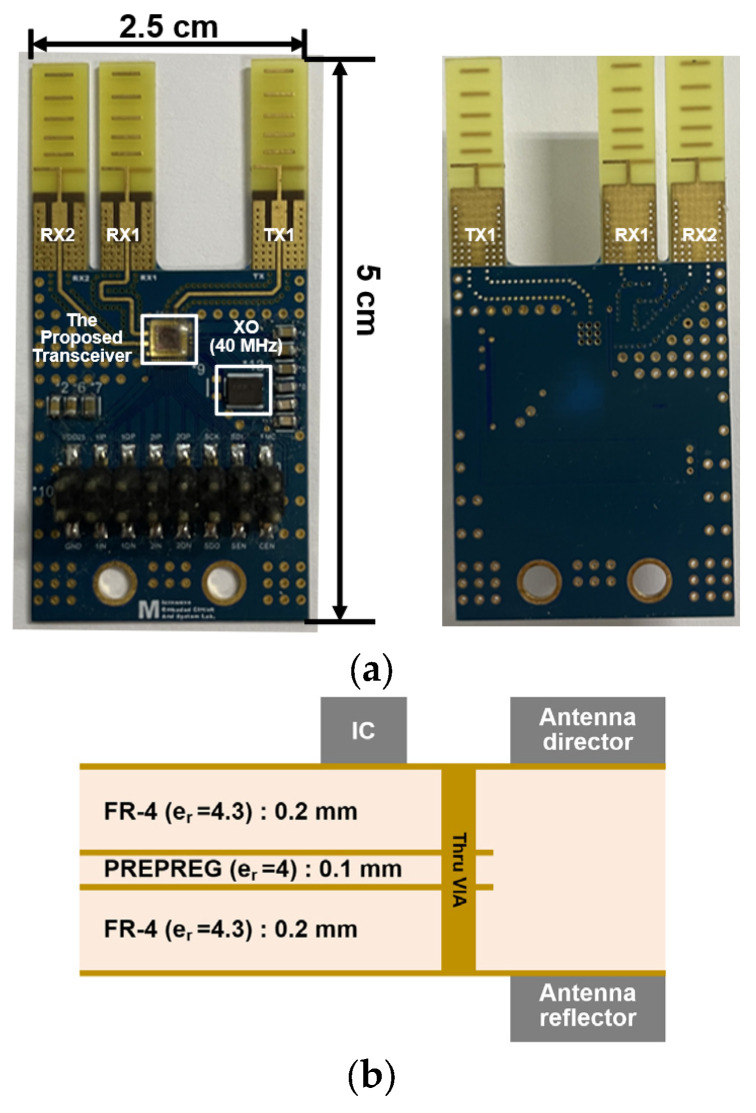
(**a**) Implemented prototype FMCW radar module using the proposed transceiver and Yagi antenna and (**b**) stack up of the radar module.

**Figure 20:**
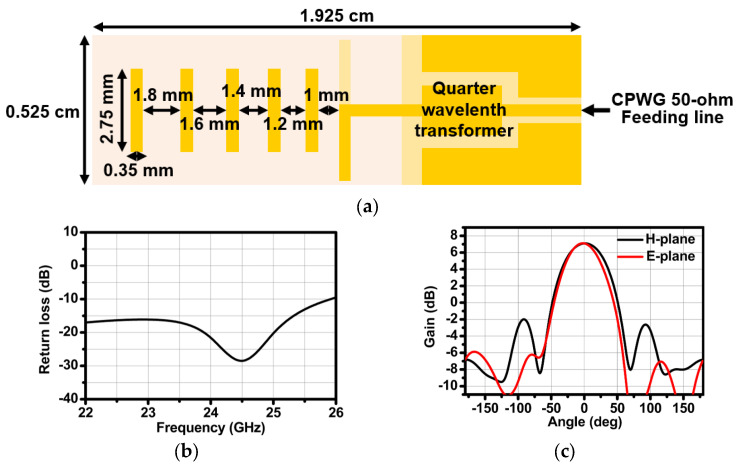
(**a**) Design configuration, (**b**) simulation results of the Yagi antenna, and (**c**) simulated H-plane and E-plane beam patterns.

Figure 19a shows the prototype FMCW radar module. The proposed transceiver was glued onto the module and gold-bonded in a chip-on-board (COB) layout. Furthermore, a 40 MHz crystal was mounted for the reference frequency. A total of three unit Yagi antennas were employed, and the distance between the two Yagi antenna for the RXs was set as ʎ/2. The prototype radar module consists of a total of 4 layers using FR-4, as shown in Figure 19b. The director of the Yagi antenna was placed on the top layer where the transceiver was glued, and the ground reflector was placed on the bottom layer. Figure 20 outlines detailed design parameters and simulation results related to the unit Yagi antenna.

**Figure 21:**
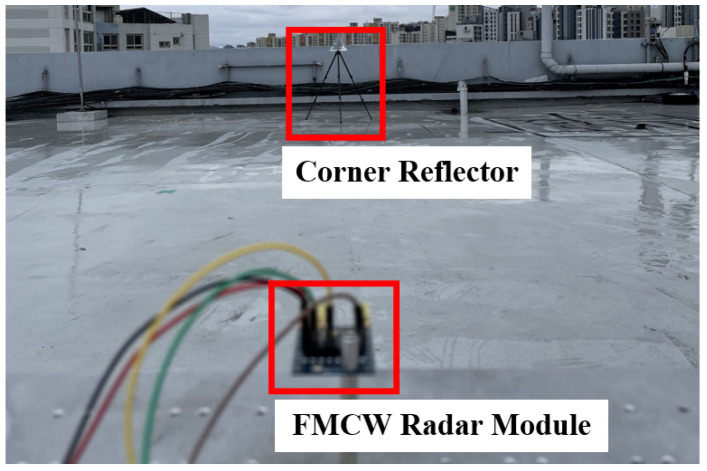
Measurement setup.

**Figure 22:**
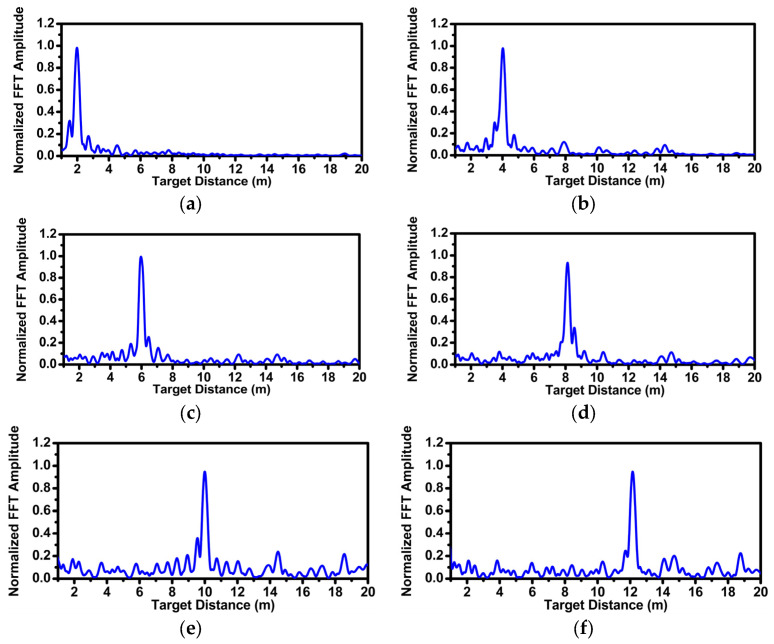
Range measurement: (**a**) 2 m, (**b**) 4 m, (**c**) 6 m, (**d**) 8 m, (**e**) 10 m, and (**f**) 12 m.

**Figure 23:**
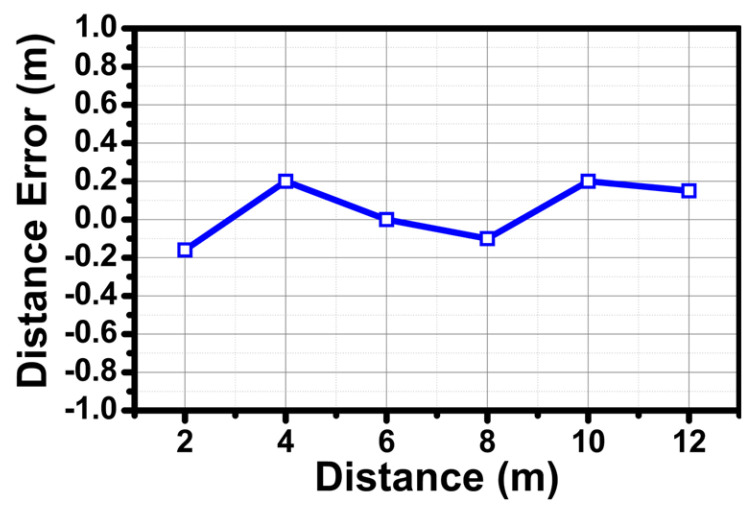
Range measurement: distance error between real distance and measured distance.

**Figure 24:**
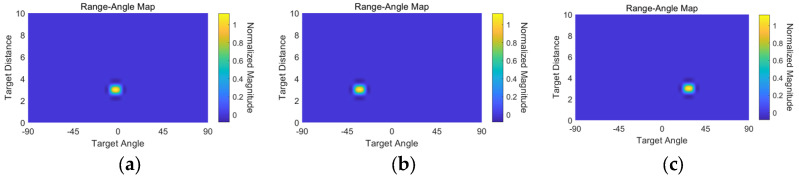
Angle measurement: (**a**) 0 degree, (**b**) −30 degree, and (**c**) 30 degree.

The unit Yagi antenna consisted of 5 directors, a quarter wavelength transformer for impedance matching, a 50 ohm feeding line with coplanar waveguide with ground (CPWG), and a ground reflector. The Yagi antenna achieved a return loss of <−20 dB within the frequency range of 24 GHz to 25 GHz, as shown in Figure 20a. Finally, the simulated H-plane and E-plane beam patterns are depicted in Figure 20b,c. The gain was about 7 dB, and the half power beamwidth (HPBW) was 69.4° in the H-plane and 59.4° in the E-plane. The radar functionality has been verified in real environments by estimating the range and angle of a target. A corner reflector with a high radar cross section (RCS) was used as a target in the real environment. As shown in Figure 21, the corner reflector was placed in front of the radar module. The distance between the radar module and the reflector was varied at 2 m intervals. The chirp waveform characteristics in the measurement are as follows: chirp bandwidth 250 MHz, Up-chirp time 50 μs, Down-chirp time 5 μs, and Hold-chirp time 0 μs. Figure 22 shows the measured results, which were obtained by performing FFT on the IF signals of receiver channel-1, and Figure 23 shows the distance error between the real distance and the measured distance. For all distances the error was measured to be below 0.2 m. Finally, for angle detection, the corner reflector was placed 3 m away from the radar module. The angle detection measurements were made by changing the angles of the corner reflector with high RCS to −30, 0, and +30 degrees. Similar to range estimation, after performing FFT on the IF signals of receiver channels 1 and 2, the phase of the only IF signal with maximum magnitude was extracted, and then the angle was estimated through the mono-pulse angle estimation method. Figure 24 shows the measurement results of angle detection. 

## 5. Conclusions

A fully integrated 1TX 2RX transceiver for a 24 GHz FMCW monopulse radar is here presented. The entire transceiver was realized with a small chip size of 4.84 mm^2^ through full integration. The FMCW synthesizer has been proposed for fast chirp generation and a reconfigurable chirp waveform. The synthesizer achieved various waveforms such as sawtooth and triangular waveform with chirp time and chirp bandwidth variation, and a phase noise of −80 dBc/Hz at the offset frequency of 100 kHz. The TX consists of a bridged T-type ATT, a DA and a PA. The PA optimized the load impedance through load pull testing and achieved an output power of 12.6 dBm up to 14.6 dBm, depending on the supply voltage of the LDR. The RX achieved a wideband gain in the front-end stage by using a magnetically coupled resonator with a low coupling factor k. The TIA and the two-stage BPF can be used to adjust the gain and cut-off frequency according to the target distance budget. The overall RX achieved high linearity characteristics and an overall maximum gain of 100 dB. The prototype FMCW radar module was developed using the proposed transceiver and Yagi antennas. The radar module has demonstrated the ability to detect the positions of targets in real environments.

## Figures and Tables

**Table 1:** Current consumption of sub blocks.

Block	Current Consumption	Unit
FMCW Synthesizer	40	mA
1-channel TX	70	
2-channel RXs	90	
Supporting Circuit, LDRs	20	

**Table 2:** Performance comparison.

Block	Unit	This Work	[5]	[6]	[7]	[8]	[9]
Technology	·	65 nm CMOS	130 nm SiGe	130 nm SiGe	130 nm CMOS	130 nm CMOS	55 nm CMOS
Frequency	GHz	24	24	24	24	24	24
The number of channel		1TX-2RX	2TX-2RX	1TX-3RX	1TX-1RX	1TX-1RX	1TX-2RX
FMCW generation	·	Built-in	External PLL	External PLL	External PLL	External PLL	Built-in
Modulation BW ^&^	MHz	250	200	·	250	250	250
Modulation Time ^&^	μs	50	150	·	2000	1000	2000
Integrated baseband	·	Yes	Yes	Yes	No	Yes	Yes
Phase Noise @ 100 kHz	dBc/Hz	−80	−111 ^+^	−100 ^+^	−105.4 ^+^	−84	−71
TX output power	dBm	13.3	5	13	−1.6	5	11
RX maximum gain	dB	100	87	60	15.3 ^^^	56	64
Die size	mm^2^	4.84	·	9	1.53	2	7.84
Current consumption	mA	220	160	·	110	·	344

^+^: The phase noise of the VCO at an offset frequency of 1 MHz. ^^^: LNA + Mixer. ^&^: Chirp parameter value when the transceiver was in FMCW mode.

## Data Availability

The data can be obtained from the authors on request.
